# Medicines availability at a Swaziland hospital and impact on patients

**DOI:** 10.4102/phcfm.v7i1.829

**Published:** 2015-09-14

**Authors:** Kholiwe Shabangu, Fatima Suleman

**Affiliations:** 1 Discipline of Pharmaceutical Sciences, School of Health Sciences, University of KwaZulu-Natal, Durban, South Africa; 2 Discipline of Pharmaceutical Sciences, School of Health Sciences, Westville Campus, University of KwaZulu-Natal, South Africa

## Abstract

**Background:**

The burden of non-communicable diseases (NCDs) in low- and middle-income countries is increasing. Where patients are expected to make increased out-of-pocket payments this can lead to treatment interruptions or non-adherence. Swaziland is no exception in this regard.

**Aim:**

The aim of the study was to investigate the availability of medicines for NCDs in a hospital and the impact of out-of-pocket spending by patients for medicines not available at the hospital.

**Setting:**

The study was conducted at Raleigh Fitkin Memorial Hospital in Manzini, Swaziland.

**Methods:**

Exit interviews to assess availability of a selected basket of medicines were conducted with 300 patients diagnosed with diabetes, hypertension or asthma. The stock status record of a basket of medicines for these conditions in 2012 was assessed at the Central Medical Stores. Results were analysed using the Statistical Package for Social Sciences version 20.0.

**Results:**

Most of the patients (*n* = 213; 71%) confirmed not receiving all of their prescribed medicines at each visit to the hospital in the past six months. On average patients spent 10–50 times more on their medicines at private pharmacies compared to user fees in the health facility. Stock-outs at the Central Medical Stores ranging from 30 days to over 180 days were recorded during the course of the assessment period (12 months), and were found to contribute to inconsistent availability of medicines in the health facility.

**Conclusion:**

Out-of-pocket expenditure is common for patients with chronic conditions using this health facility, which suggests the possibility of patients defaulting on treatment due to lack of affordability.

## Abstract (fr)

**Disponibilité des médicaments dans un hôpital du Swaziland et son impact sur les malades.**

**Contexte:**

Le fardeau des maladies non transmissibles (MNT) dans les pays à revenus faibles et moyens est en augmentation. Quand les patients doivent débourser des sommes importantes il en résulte des interruptions ou la non-adhésion au traitement. Le Swaziland ne fait pas exception à la règle.

**Objectif:**

Le but de l’étude était d’examiner la disponibilité des médicaments pour les MNT dans un hôpital et l’impact des frais à la charge des patients pour les médicaments non disponibles à l’hôpital.

**Lieu:**

L’étude a été faite au Raleigh Fitkin Memorial Hospital de Manzini, au Swaziland.

**Méthodes:**

Des entretiens de sortie ont eu lieu avec 300 patients souffrant de diabète, pour évaluer la disponibilité d’un panier sélectionné de médicaments. L’état des stocks d’un panier de médicaments pour ces maladies en 2012 a été évalué au Dépôt médical central. Les résultats ont été analysés en utilisant le logiciel « Statistical Package for Social Sciences », version 20.0.

**Résultats:**

La plupart des patients (*n* = 213; 71%) ont admis qu’ils n’avaient pas reçu tous les médicaments prescrits à chaque visite à l’hôpital au cours des six derniers mois. En moyenne les patients ont dépensé de 10 à 50 fois plus pour leurs médicaments dans les pharmacies privées, comparé aux frais d’utilisation dans les services de santé. Au cours de la période d’évaluation (12 mois) on a constaté des ruptures de stock de 30 à 180 jours dans le Dépôt médical central, ce qui a contribué à la disponibilité irrégulière des médicaments dans le service de santé.

**Conclusion:**

Les frais à la charge des patients sont courants pour les patients souffrant de maladies chroniques utilisant ce service de santé, ce qui peut amener ceux-ci à interrompre le traitement en raison de son coût élevé.

## Introduction

Non-communicable diseases (NCDs) have emerged as a clear threat not only to human health but also to development and economic growth globally. The World Economic Forum estimated that 63% of all deaths occurring globally are caused by NCDs. ^1^ Moreover, NCDs are no longer considered only to be diseases of the affluent, but also affect large number of patients in developing countries. Eighty percent of deaths from NCDs are said to be occurring in low- and middle-income countries, which poses a real challenge to health systems in these countries.^1^ Furthermore, globally NCDs are increasingly becoming common amongst all age groups, with a quarter of all related deaths occurring amongst people below the age of 60 years.^2^ In addition NCDs account for over 48% of healthy life-years lost globally, compared to 40% for communicable diseases and 1% for injuries.^3^

In Swaziland both communicable diseases and NCDs continue to be a major challenge.^4^ The death rate due to NCDs stood at 4.9/1000 in 2008.^5^ NCDs have received inadequate attention in Swaziland, given the serious double burden of disease that prevails in the country: communicable diseases and HIV infection and AIDS.^6^

The Swaziland Government is solely responsible for procurement and storage of essential medicines for the country’s public health facilities. This has not been without challenges. The Swaziland National Pharmaceutical Policy states that the current warehouse can hold six months’ supply of medicines and medical supplies that it procures, stores and distributes to all Government hospitals, mission hospitals, parastatals, clinics and health centres. It is further claimed that this restricted capacity places a burden on the logistics system, with repeated ordering of inventory, and contributes to frequent stock-outs of prescription medicines at health facilities.^7^

User fees of 10 Swazi Lilangeni (SZL) (US $1.13) for hospitals and SZL 5 (US $0.56) (Central Bank of Swaziland Exchange rate: US $1 = SZL10 as at 15 November 2013) are applied at clinics, which includes consultation and the supply of medicines (when available).^7^ When medicines for the treatment of NCDs are out of stock at health facilities and patients are required to purchase them from private pharmacies this would constitute a major barrier to access.

The aim of the study was to investigate the availability of a basket of medicines for selected NCD conditions in a health facility in Swaziland and the impact of out-of-pocket spending by patients for medicines that are not available at this hospital.

## Research methods and design

### Study design

The study was a prospective cross-sectional survey that evaluated the impact on patients on chronic medication in the event that their prescribed medicines for asthma, diabetes or hypertension were out of stock at Raleigh Fitkin Memorial Hospital (RFMH) in Manzini, Swaziland.

### Setting

The study took place at RFMH, a 350-bed regional referral hospital situated in Manzini, the hub of Swaziland. The hospital attends to an average of approximately 200 000 patients per year (in- and outpatients). Approximately 40% of patients seen at RFMH have asthma, diabetes or hypertension. The number of outpatients that receive medications from the pharmacy averages 750 patients a day, as not all outpatients visit the hospital pharmacy. An average of 200 patients is admitted into the hospital wards per day, which accounts for an average of 57% bed occupancy at any given time (Human Resources Offices of RFMH 2013, personal communication, March).

### Study population and sampling strategy

The study population comprised all patients seen for the three NCDs (diabetes, asthma and hypertension) in the facility, and who had a single diagnosis of one of these three conditions. On average a total of 1111 patients attended RFMH every month for consultations for asthma, diabetes orhypertension (Human Resources Offices of RFMH 2013, personal communication, March).

A statistical formula was used to calculate the number of patients to be interviewed based on the monthly statistics of the hospital,^8^ with a 5% margin of error, a 95% confidence interval and a 50% response distribution. Setting the response distribution at 50% is the most conservative assumption when one is not sure what to expect the results for each question to be. By using 50% the largest sample size is calculated.

Based on the calculation a sample of 300 patients with the chronic conditions of diabetes, hypertension or asthma were selected at their point of exit from the pharmacy and interviewed using a questionnaire. A quantitative coded structured questionnaire with some open-ended questions on patients’ perception of costs of medicines that were not available at the hospital was used for exit interviews with patients that fulfilled the inclusion criterion. The questionnaire was divided into three sections: the demographics section, including patients’ employment status; a section on patients’ experiences of stock-outs of medicines; and a section on out-of-pocket costs of buying the medicines. Both SiSwati and English versions of the questionnaires were available to allow patients to choose their preferred language of interview.

At Central Medical Stores (CMS) stock status data for the ten selected medicines making up the chosen basket, based on the current treatment for these conditions in the existing national Standard Treatment Guidelines and the Essential Medicines List at the time of the study, were used to check availability of the medicines and the time it took for them to become available at RFMH after receipt for the period July 2012 to June 2013.

### Data collection

Data were collected from patients who had come to fetch their medicines from the pharmacy through exit interviews. The interviews were done after patients had collected their prescribed medicines at the pharmacy, and were conducted by two second-year pharmacy students from the Southern Africa Nazarene University after the prescriptions had been pre-selected by the dispensing personnel (i.e. pharmacy technicians and pharmacists). The selection of medical files was conducted by the professional staff based on the diagnosis of the patient (those having any of the three NCDs under study, with a diagnosis as presented in the clinical notes for more than six months, were eligible). The data collectors then randomly selected adult patients (18 years of age and over) from these files. The data collection was carried out over a period of six weeks, from 23 August 2013 to 4 October 2013. Only patients who were refilling their medications at RFMH during the study period were eligible.

The questionnaire was used to: (1) determine if patients had been exposed to out-of-stock of medicines for their chronic conditions in this facility; (2) establish the coping strategies they were using in order to access their medicines in the event that they were out of stock in the facility; (3) identify the consequences of the unavailability of medicines in the facility to patients and their immediate families; and (4) establish the affordability or otherwise of purchasing medicines out of pocket and any complications experienced due to medicines not being available in the facility.

Data collection at CMS was done through the Senior Pharmacist, who provided the stock status records for the selected medicines from July 2012 to June 2013 to the principal researcher. The records were used to calculate the number of days each medicine was out of stock at the CMS (from the date it was recorded as stock-out) and how soon it was made available to RFMH after the date of receipt (time between getting the stock and processing the stock to RFMH).

### Data analysis

Data were categorised, coded and entered first into an Excel spreadsheet and later imported into the Statistical Package for Social Sciences (version 20.0), where descriptive statistical analysis was conducted. The open-ended questions were coded and categorised into themes.

### Ethical considerations

Ethical clearance to perform the study was obtained from the University of KwaZulu-Natal Humanities and Social Sciences Research Ethics Committee (HSS/0620/013M) and the Ministry of Heath Ethics Committee in Swaziland (MH/599C/FWA00015267). Patients provided informed consent and participation was voluntary.

## Results

### Demographic information

The largest age group in the sample was that aged 50–64 years (*n* = 128; 42.7%), and most of the patients with chronic conditions were females (*n* = 215; 71.7%). The results showed that whilst the majority of patients (*n* = 249; 83%) had received some form of education, only a few (*n* = 51; 17%) had reached tertiary education. The rest (*n* = 51; 17%) had never been to school. Most of the patients were married (*n* = 154; 51.3%).

The rate of unemployment amongst these patients was very high (*n* = 118; 39.3%); only 25% (*n* = 75) were employed, with those who were self-employed accounting for 22.7% (*n* = 68).

Results on income distribution (see Table 1) showed that 44.3% (*n* = 133) of the interviewed patients had a monthly income of less than SZL 500 (US $50). Only 9.7% (*n* = 29) of the patients had an income above SZL 5000 (US $500) per month.

**TABLE 1:** Patients’ monthly income distribution (SZL).

Monthly income (SZL)	Number of patients	%
≤500	133	44.3
501–1000	67	22.3
1001–2000	48	16
2001–5000	23	7.7
˃ 5000	29	9.7

SZL, Swazi Lilangeni.

Central Bank of Swaziland Exchange rate: US $1 = SZL10 as at 15 November 2013.

### Health status and health-seeking behaviour

Of the participants in the study, most were hypertensive (41%; *n* = 123) and diabetic (39.3%; *n* = 118). Fifty nine (19.7%) of these patients were asthmatic. Most of the patients had been diagnosed with their conditions for more than two years (*n* = 205; 68.3%). Although most of the patients (*n* = 243; 81%) reported returning on a monthly basis to refill prescriptions, the two other options provided were reported by 3% (once every 2 months, *n* = 9) and 16% (only when feeling sick, *n* = 48). Patients who failed to come for their refills on a monthly basis were asked why they failed to adhere to their appointments. In response, 38.2% (*n* = 21) said they did not have money for transport to the facility, 29.1% (*n* = 16) said they did not find it necessary to do refills on a monthly basis, 20% (*n* = 11) said they were not informed that they were supposed to come to the hospital on a monthly basis, and 12.7% (*n* = 7) said they did not have money to pay for a consultation (user fees). The participants’ monthly income distribution is indicated in Table 1.

### Medicine availability and coping strategies of patients

Results showed that only 24.7% (*n* = 74) of interviewed patients said they always got all their prescribed medicines at the hospital’s dispensary, as indicated in Table 2. Further analysis of these patients showed that 13.5% (*n* = 10) of them had asthma, 48.6% (*n* = 36) had diabetes and 37.8% (*n* = 28) had hypertension. Seventy-nine per cent (*n* = 237) of the patients reported having received all their prescribed medication on the day they were interviewed; 13% (*n* = 30) of these were asthma patients, 48% (*n* = 114) diabetic patients and 39% (*n* = 93) hypertension patients. However, 16.7% (*n* = 50) of the patients did not receive all their prescribed medicines on the day they were interviewed, and 4.3% (*n* = 13) were not sure if they had received all of their prescribed medicines or not, which could indicate either their lack of understanding of their treatment or a breakdown in communication and counselling by health professionals in the facility treating them. Most (94%) of the patients were referred to a private pharmacy in the event of medicines stock-out at the health facility.

**TABLE 2:** Medicine availability and coping strategy of patients (*n* = 300).

Variables	Frequencies	%
**Always received prescribed medicines in the facility**		
Yes	74	24.7
No	213	71
Don’t know	13	4.3
**Frequency of not receiving prescribed medicines in the past six months**		
Once	94	31.3
Twice	29	9.7
More than three times	29	9.7
Not sure	148	49.3
**Received all prescribed medicines on the day of interview**		
Yes	237	79
No	50	16.7
Not sure	13	4.3
**Proportion of prescribed medicines received**		
None (0% – 50%)	25	8.3
Partly (51% – 80%)	12	4
Mostly (81% – 99%)	26	8.7
Completely (100%)	237	79
**Instruction conveyed by dispensing personnel for medicines not available in the facility**		
Returned to the doctor	18	6
Referred to private community pharmacy	282	94
**Turnaround time for buying medicines at the chemist**		
As soon as I leave the facility	119	39.7
Whenever, after getting money	152	50.7
When I go to town as there is no chemist in the community	8	2.7
When I get paid	11	3.7
When I feel sick	10	3.3
**Out-of-pocket expenditure by patients in private pharmacies**		
˂ SZL 100	191	63.9
SZL 100–SZL 300	86	28.8
SZL 301–SZL 500	7	2.3
˃ SZL 500	15	5
**Health insurance cover**		
Yes	15	5
No	285	95
**Health insurance cost incurred**		
˂ SZL 1000	5	1.7
˃ SZL 1000	4	1.3
Not sure, paid by spouse	6	2
**Number of family members supported **		
1	82	27.3
2	37	12.3
3	18	6
˃ 5	27	9
None	136	45.3

It is important to note that half of the patients who were interviewed (50.7%) indicated that they only bought their medicines when they had money to do so. Patients estimated what they paid for medicines at the pharmacy, with 63.7% (*n* = 191) paying less than SZL 100 (US $10) for their medication at private pharmacies. About 29% (*n* = 86) of the patients indicated that they were paying between SZL 100 and SZL 300 (US $10–US $30), whilst 2.3% (*n* = 7) said they were paying between SZL 301 and SZL 500 (US $30 – US $50) for their medication at a private pharmacy. Five per cent (*n* = 15) were paying more than SZL 500 (US $50) when buying their medication out of pocket in the event of stock-out at the facility for that month. Only 5% of the patients had health insurance cover.

### Stock availability at the Central Medical Stores

**FIGURE 1:**
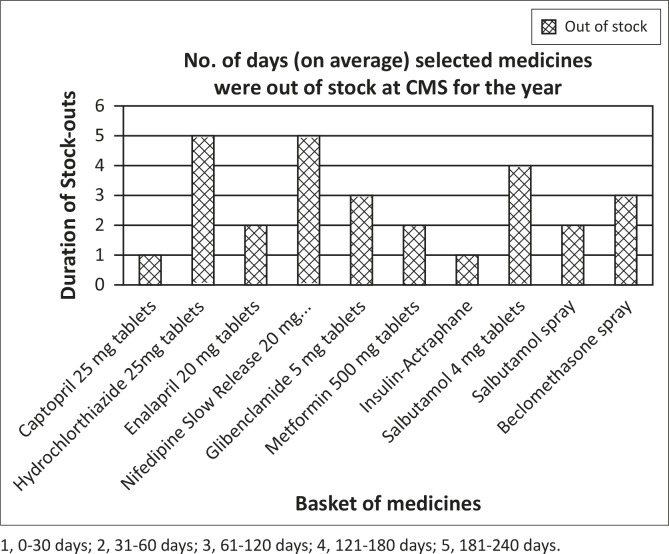
Availability of the selected basket of medicines at the CMS during the period June 2012 to July 2013.

It is evident from Figure 1 that on average, hypertensive medications were least available in the 12-month period when compared to the other groups, followed by asthma medication. Diabetes medication was more available when compared to the other groups of medications. The results also showed that on average medicines for the selected NCDs were out of stock at the CMS for between two and four months (31–120 days) out of the year.

In terms of turnaround time, most (70%, *n* = 7) of the medicines were issued to the health facility within less than 15 days after receipt by the CMS (based on back orders), namely beclomethasone spray, enalapril 20 mg, Actraphane insulin, captopril 25 mg, metformin 500 mg and nifedipine slow release 20 mg tablets. This is the lead time stipulated in the Pharmaceutical Standard Operational Procedures of the country for ordering of medicines by health facilities. Salbutamol spray was issued between 25 and 30 days after receipt, whilst glibenclamide was issued between 35 and 40 days after receipt at the CMS. Data on hydrochlorothiazide 25 mg tablets was unavailable at the time of data collection, hence one could not gather whether this medicine was issued to the health facility or not.

## Discussion

This study showed that in the sample population NCDs were more common in those above 35 years of age and in females. This result is similar to that found in sample populations in other studies, that reported that mostly females were affected by chronic conditions compared to males.^9^ This may also be due to the generally high rate of health-seeking behaviour amongst the female population.^10^

Almost half of those who were interviewed (44%) earned a monthly income of less than SZL 500 (US $50), which means that patients may have to forgo other household necessities in order to access medications or health services to manage their conditions. Although these patients were not asked how they were financing their health expenditure, a number of studies have shown that patients with chronic conditions will use a variety of ways to finance their health expenditure: some would sell their assets or borrow money.^11^ Outpatient treatment costs have been implicated in catastrophic health expenditure in another setting – India.^12^

Just over half (50.7%) of the patients reported they had not received all of the medicines prescribed when presenting for a refill in the six months prior to the survey. It was notable though that the balance were ‘Not sure’, and no patient reported always receiving all their medicines at every refill visit. This factor could result in a number of outcomes in the care of patients, which would include complication of their conditions due to foregoing therapy (as they might be unable to afford private pharmacy prices), hospitalisation, expensive treatment for complications and loss of income due to hospitalisation.

Although some patients were able to buy the out-of-stock medicines at private pharmacies immediately after leaving the facility, a significant number (60.4%) could not because of financial constraints. Out-of-pocket amounts spent on medicines by these patients were estimated to be between 10 times and 50 times more than the user fees (SZL 10) charged to access healthcare services in any health facility in the country.

A 2004 study amongst 875 adults with diabetes in the United States of America showed that out-of-pocket medication costs posed a significant problem, affecting their adherence as well as other aspects of their life.^13^ Patients’ decision-making process would need to be researched further in terms of priority items that income is expended upon, and the position that medicines play in that process. Health systems may be failing these patients, and the result could be an additional burden on healthcare resources when they present with complications or in advanced stages of their conditions.

The stock availability of the selected medicines at the CMS varied. Some medicines were out of stock for a period of less than 30 days a year (captropril and Actraphane insulin), whilst others were out of stock for more than six months (hydrochlorothiazide and nifedipine slow release 20 mg). The latter two are also used in the treatment of cardiovascular conditions, and the severity of the out-of-stock situation may actually be more extreme than is reflected in this exploratory research. The turnaround time of medicines upon receipt at the CMS to the RFMH was acceptable, as it varied from one to 30 days.

This evidence demonstrates that the Ministry of Health needs to assess and improve the availability of medicines in health facilities if required, in order to negate a greater impact on healthcare resources at a later stage of the patient’s condition. Access to essential health care and medicines is a basic human right, and at the least the essential medicines for primary care should be available at all times.

### Limitations

The study setting is not representative, as patients were sampled from one facility in one region, and hence the results cannot be generalised to other public health facilities in the country. A study with a larger target group would need to be done in more public health facilities to see if the responses are the same and to be able to generalise results.

The cost of medicines in the private pharmacies is not based on actual costs on the day of the interview; as such, the cost of medication at the private pharmacies could not be verified, and the costs are estimates provided by patients. A pricing study should be conducted on the basket of medicines to ascertain the price and availability of these essential medicines at private pharmacies.

Costs to patients are not exhaustive. Whilst the direct out-of-pocket costs of buying the medicines could be estimated, these were a small fraction of the total costs incurred by patients and their families in their efforts to access treatment for their conditions. Inclusion of indirect costs like transport and loss of income would provide a better picture of the financial catastrophe which patients face in order to access health care.

### Recommendations

Research on coping strategies for patients with NCDs should be conducted on a larger population and include more public health facilities in order to ascertain how they are coping with the burden of out-of-pocket expenditure when accessing their medicines at private pharmacies. A pricing survey should be conducted to determine actual costs to patients of essential medicines within the private sector. Models of public-private partnerships to make essential medicines available to patients in the event of stock-outs should be developed and explored. Pharmacists in the CMS should be evaluated to determine their skills in medicine supply management, and reasons for stock-outs need to be clarified.

## Conclusion

NCDs have shown to be a growing threat in diseases that increase morbidity and mortality in low- and middle-income countries. Proper management of these conditions and uninterrupted availability of medicines will result in a significant reduction of complications and the need for expensive treatment and improved health outcomes in NCD patients in the country.

This exploratory study has suggested that availability of essential medicines for three NCDs at a hospital in Swaziland is problematic, and that patients face a difficult choice when required to access these medicines from the private sector. Logistic supply systems for medicines need to be examined to determine where the bottlenecks are and to overcome these in order to provide an uninterrupted supply of medicines to patients.
